# BACKWARD WALKING SPEED: A NOVEL MOBILITY MEASURE FOR YOUNGER AND OLDER ADULTS

**DOI:** 10.1093/geroni/igad104.2681

**Published:** 2023-12-21

**Authors:** Braden Popelsky, Kelly Ylitalo, Samuel Hockett

**Affiliations:** Baylor University, Waco, Texas, United States; Baylor University, Waco, Texas, United States; Baylor University, Waco, Texas, United States

## Abstract

Mobility is fundamental to healthy aging. Forward walking speed (FWS) is a well-established measure of mobility, physical functioning, and fall risk but fewer studies have assessed backward walking speed (BWS). This study explored the relationship between FWS, BWS, and fall-related factors. Independently ambulatory, community-dwelling young adults (n=20; age: 22.9 years ±1.6) and older adults (n=19; age: 70.2 years ±6.2) completed forward and backward walking assessments on a 3.9 meter long instrumented pressure mat. Paper surveys assessed sociodemographic characteristics, fall history, and fear of falling using the Falls Efficacy Scale – International (FES). FWS, BWS, and directional difference percent change ((FWS-BWS/FWS)*100) were compared using independent sample t-tests, and simple linear regression was used to model the relationship between mobility measures and fear of falling. In the total sample (n=39), 23% of adults reported a fall in the previous 12 months. Compared to older adults, young adults exhibited significantly faster FWS (138.3 cm/sec ± 16.5 vs. 119.3 cm/sec ±23.4; p=0.006) and BWS (82.6 cm/sec ±18.2 vs. 63.8 cm/sec ±20.7; p=0.005). Males displayed a significantly lower mean directional difference percent change (39.4 ±11.4 vs. 47.4 ±11.1; p=0.034), than females. Among older adults, BWS was strongly and negatively correlated with FES scores (r=-0.58; p=0.009). BWS explained a greater proportion of variance in fear of falling compared to FWS (33.93% vs. 24.84%). BWS and directional difference in walking speed may be novel measures of physical functioning. Prospective studies should consider assessing BWS with future fall risk among older adults.

